# The Spatial and Temporal Distribution of Bigeye Tuna and Yellowfin Tuna in the Northwest Indian Ocean and Their Relationship with Environmental Factors

**DOI:** 10.3390/ani16020282

**Published:** 2026-01-16

**Authors:** Guoqing Zhao, Hanfeng Zheng, Chao Li, Yongchuang Shi, Fengyuan Shen, Hewei Liu, Jialiang Yang, Ziniu Li, Zhi Zhu, Lingzhi Li

**Affiliations:** 1 East China Sea Fisheries Research Institute, Chinese Academy of Fishery Sciences, Shanghai 200090, China; zgq617717@163.com (G.Z.); zhenghf@ecsf.ac.cn (H.Z.); bhrock05@sina.com (C.L.); syc13052326091@163.com (Y.S.); fyshen0817@126.com (F.S.); hwliu77@126.com (H.L.); yangjl@eastfishery.ac.cn (J.Y.); lizn@ecsf.ac.cn (Z.L.); dccglc@ecsf.ac.cn (Z.Z.); 2 Key Laboratory of Oceanic and Polar Fisheries, Ministry of Agriculture and Rural Affairs, East China Sea Fisheries Research Institute, Chinese Academy of Fishery Sciences, Shanghai 200090, China

**Keywords:** bigeye tuna, yellowfin tuna, resource abundance, hotspot analysis, environmental factors, Northwest Indian Ocean

## Abstract

The Northwestern Indian Ocean (NWIO) serves as a primary fishing ground for tuna longline fisheries, with bigeye tuna (*Thunnus obesus*) and yellowfin tuna (*Thunnus albacares*) constituting the main target species. Investigating their spatiotemporal distribution and relationship with environmental factors is of significant importance for fishery management and fishing. This study analyzed and compared the distribution patterns and environmental preferences of these two species across different depth layers, based on fisheries scientific survey data collected during the 2023/2024 and 2024/2025 fishing seasons. Key findings include: The hook rate in 2023/2024 was higher than in 2024/2025, and the hook rate for *T. obesus* exceeded that of *T. albacares*. *T. obesus* were predominantly concentrated within 63° E–69° E and 7° N–9° N, while *T. albacares* exhibited a broader yet more dispersed distribution range. *T. obesus* primarily occupied depth layers of 130–140 m (12.20%), 180–190 m (9.76%), and 270–280 m (9.76%). *T. albacares* were mainly found at 110–120 m (15%), 140–150 m (15%), and 200–210 m (15%). Both species exhibit distinct spatial clustering patterns, and their hotspot distribution areas are, respectively, 63° E–69° E, 5° N–10° N and 64° E–68° E, 0° N–4° N. Correlation analysis revealed significant relationships between *T. obesus* distribution and latitude, zooplankton abundance, water temperature at various depths, and chlorophyll a concentration. Our research provides reference for understanding the distribution of *T. obesus* and *T. albacares* across different water layers and their habitat preferences, laying a scientific foundation for achieving sustainable utilization of both species.

## Simple Summary

Bigeye tuna (*Thunnus obesus*) and yellowfin tuna (*Thunnus albacares*) are the primary target species of longline fisheries in the northwestern Indian Ocean (NWIO). Their population distribution patterns and environmental preferences are critical to fisheries production, resource management, and the elucidation of their migratory behaviors. This study used scientific survey data from two research cruises to comparatively analyze the spatial distribution, vertical distribution, and environmental preferences of the two species. It was confirmed that *T. obesus* occupies deeper and broader vertical habitats, whereas *T. albacares* exhibits a wider horizontal distribution range. The distribution hotspots of the two species also differ significantly, with *T. albacares* showing a stronger preference for warmer waters than *T. obesus*. Environmental factors across different water layers exert a substantial influence on the distributions of both species. Our findings provide crucial insights for further investigations into the environmental preferences and underlying migratory mechanisms of these tuna species, particularly with respect to their transoceanic connectivity.

## 1. Introduction

The Indian Ocean is the world’s second-largest tuna fishing region, accounting for approximately 20% of the global total tuna catch [1,2]. It is a critical component of the global tuna fishery and is recognized as one of the world’s major high-seas tuna fishing grounds [3]. Among target species, bigeye tuna (*Thunnus obesus*) and yellowfin tuna (*Thunnus albacares*) are the primary catches of the Indian Ocean longline tuna fishery, and their resource status and spatiotemporal distribution characteristics are critical to the sustainable development of fisheries production and the stability of marine ecosystems. In recent years, the fishing mortality rate of *T. albacares* in the Indian Ocean has continued to rise, and assessment results over the past decade have further shown that this species is under increasing overfishing pressure [4]. *T. obesus* is a slow-growing species [5], and over the past 20 years, approximately 40% of its global distribution areas have been subject to overfishing [6]. Globally, its population biomass is estimated to have decreased by 29–32%, with reductions of 40% recorded in the Atlantic and Indian Oceans [6]. Against this backdrop, the International Union for Conservation of Nature (IUCN) has listed *T. obesus* as a Vulnerable species [6].

*T. obesus* and *T. albacares* are highly migratory, epipelagic, warm-water species widely distributed in tropical and subtropical oceans [7]. They exhibit an extremely wide distribution range, primarily occurring in the Pacific and Indian Oceans, and are regulated by multiple international fisheries organizations [1,2]. Their abundance and spatiotemporal distribution are significantly influenced by physical and biological environmental parameters [8]. Globally, sea surface temperature (SST) has been identified as one of the most critical environmental variables driving the habitat suitability of tropical tunas, and the most significant predictive variable for both *T. obesus* and *T. albacares* [8,9]. Additionally, subsurface temperature, chlorophyll-a concentration, dissolved oxygen (DO), and sea surface height (SSH) are recognized as important environmental factors affecting tuna habitats [9,10,11,12]. To better understand the spatiotemporal distribution of these species, numerous studies have been conducted on their habitats. However, the data used are predominantly derived from commercial fisheries and remotely sensed and derived data [8,9,10,11,12], which may introduce significant errors due to inherent biases. The vertical distribution of tunas is a key focus for fisheries producers and managers, and researchers have extensively investigated the effects of different depth layers on catch rates [9,10,13,14]. *T. obesus* and *T. albacares* exhibit highly significant differences in their vertical distribution, with the former displaying more pronounced diurnal vertical migration patterns [15,16]. Studies have demonstrated that *T. obesus* typically remain in cooler, deeper waters during the daytime, while migrating to warmer, shallower waters at night, and they can tolerate temperature differences of up to 20 °C [15]. The blood of *T. obesus* has evolved a unique characteristic, as its hemoglobin passes through the vascular countercurrent heat exchanger, its oxygen affinity decreases significantly with increasing temperature [17,18]. This distinctive blood trait requires them to maintain a relatively high muscle temperature, which is considered the primary driver behind their frequent upward migration to warm surface waters [17,18]. In contrast, *T. albacares* are unable to meet the increased oxygen metabolic demand during activities such as foraging and predator avoidance by elevating heart rate and cardiac output [19], suggesting that the influence of ambient temperature on cardiac function restricts their vertical movement [15]. Consequently, *T. albacares* primarily inhabit surface waters with relatively uniform temperatures, and the temperature variation they experience during vertical migration generally does not exceed 8 °C [15].

Understanding the environmental preferences of tuna is crucial for investigating their habitats, particularly when considering interspecific differences between coexisting species in the same region and the vertical variations in environmental factors, as this will support the implementation of commercial production activities and conservation measures. However, most existing studies on the relationship between tuna resources and environmental factors rely largely on relatively coarse fisheries production data and remotely sensed environmental data, whereas research utilizing in situ fisheries scientific survey data for analysis remains scarce. In addition, previous studies have demonstrated that *T. obesus* and *T. albacares* exhibit distinct differences in their active water layers [20,21] and possess divergent environmental preferences [22]. This study addresses the issue of data inaccuracies prevalent in prior research by conducting in situ measurements of environmental data across multiple water layers, instead of relying on data inversion methods. It further analyzed the marine environmental preferences of *T. obesus* and *T. albacares* using multi-layer environmental datasets and fisheries scientific survey data, which serve as a valuable supplement to advancing the understanding of habitat preferences in these two tuna species. Based on fisheries scientific surveys conducted during the 2023/2024 and 2024/2025 cruises, this research focuses on analyzing the spatiotemporal distribution of resource abundance of *T. obesus* and *T. albacares*, as well as their correlations with environmental factors across various water layers, using in situ collected catch data, hook depth records, and environmental datasets. Findings from this study can provide theoretical guidance for elucidating the distribution patterns of *T. obesus* and *T. albacares*, rationally adjusting the hook depth of longline fisheries, conducting fishery forecasting, and implementing science-based fisheries management strategies.

## 2. Materials and Methods

### 2.1. Surveys

**Figure 1:**
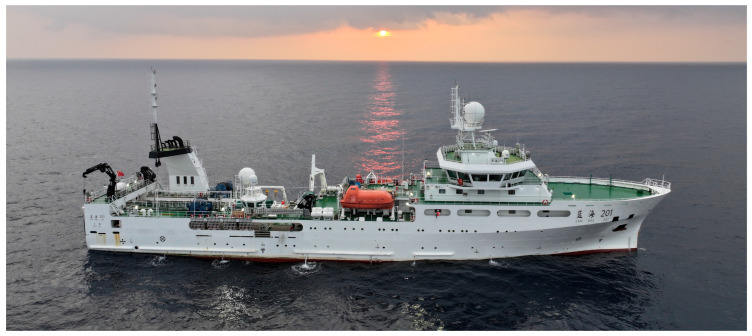
The photo of Lanhai 201.

The survey was conducted aboard the “Lanhai 201” (Figure 1), a large integrated marine fisheries research vessel with an overall length of 84.50 m, a gross tonnage of 2883 tons, and a full-load displacement of 3289.4 tons. The longline fishing gear employed in the survey consisted of main lines (nylon monofilaments), branch lines (nylon monofilaments), float lines (polyester ropes), floats (ABS injection-molded floats and circular foam floats), fishing hooks, lead pellets, and figure-eight connectors. The length of each float line was 35 m, and that of each branch line was 32 m, with an interval of 50 m between adjacent branch lines. A single fishing hook was attached to each branch line, meaning that the theoretical number of hooks between two adjacent floats was 16. The hook-setting vessel speed was adjusted according to wind speed and ocean current conditions, with the speed ranging from 5.0 to 8.2 knots and an average speed of 6.5 knots.

**Figure 2:**
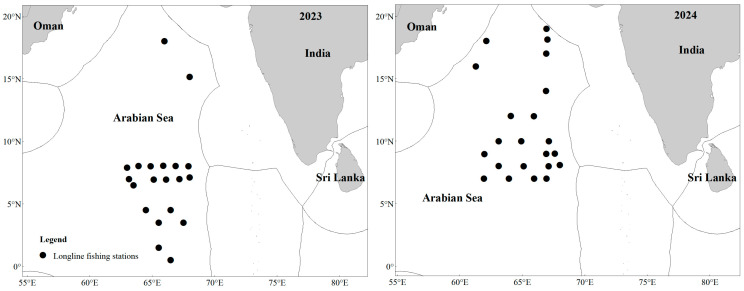
Longline fishing survey stations in the NWIO.

This study utilized data from two survey cruises conducted in the high seas of the NWIO: the 2023/2024 cruise (18 December 2023 to 5 February 2024) with 20 survey stations, and the 2024/2025 cruise (5 December 2024 to 26 January 2025) with 22 survey stations (Figure 2). Hooks were deployed at 3:00 AM local time and retrieved between 12:00 PM and 2:00 PM daily. It should be noted that the need to avoid encounters with Somali pirates led to discrepancies in the sampling stations between the two cruises. While this resulted in variations in the initial planned survey coverage, it also served to expand the overall spatial extent of the survey area.

### 2.2. Data Collection

During the survey, operational information for each hook-setting and retrieval was recorded, including the total number of hooks, hooks between floats, line deployment speed, vessel speed, and position. All catches were sampled, identified to species, photographed, and their hook positions documented.

On one selected mainline (with 16 branch lines), depth recorders (RBRsolo3D model) were attached to hooks on the 1st (shallowest), 3rd, 5th, 7th, and 9th (deepest) branch lines to measure hook depths. Using the catenary line theory, the depths of other hooks were extrapolated from these five measured nodes. A Seabird 911 plus profiler measured seawater temperature (ST), salinity (Sal) and chlorophyll-a concentration (Chl-a) at multiple depths (5 m, 25 m, 50 m, 75 m, 100 m, 150 m, 200 m, and 300 m). When conducting the correlation analysis, we directly paired the CPUE data at each sampling station with the corresponding environmental factors across different water layers at the same station, in order to identify the environmental variables that significantly affect CPUE. Concurrently, zooplankton (Abuzoo) and phytoplankton (Abuphy) abundances were analyzed in the laboratory, with all methods strictly following Marine Survey Specifications [23].

### 2.3. Data Analysis

#### 2.3.1. CPUE

Catch per unit effort (CPUE) is a key indicator for assessing fishery resource abundance, widely used in fisheries research [24]. Following the standard representation method for tuna longline fisheries, CPUE (ind/10^3^ hooks) is quantified as the number of catch per thousand hooks, with the calculation formula as follows:

(1) $$\mathrm{CPUE}\;=\;\frac{C}{H}\times 1000$$

where *C* represents the catch quantity (number of fish caught) of the target species at a survey station, while *H* denotes the actual number of hooks deployed at the corresponding station. The CPUE values for *T. obesus* and *T. albacares* are specifically expressed as CPUEdy and CPUEhq, respectively.

#### 2.3.2. Calculation of Theoretical Hook Depth

Theoretically, under conditions without external environmental influences, the mainline between two floats assumes a catenary shape. Hooks on the symmetric side of the catenary between the two floats are numbered from shallowest to deepest. Assuming the catenary shape between any two floats remains identical and the float spacing remains constant during operations, the water depth of each hook can be predicted using the theoretical depth calculation method for longline fishing [25]. The calculation formula is as follows:

(2) $$D_{j}=F+B+\frac{L}{2}\left[\sqrt{1+cot^{2}\theta }-\sqrt{{\left(2\cdot \frac{j}{N+1}\right)}^{2}+cot^{2}\theta }\right]$$

(3) $$H_{j}=\frac{L}{2\tan\theta }sinh^{-1}(tan\theta \cdot \frac{2j-N-1}{N+1})+\frac{H}{2}$$

(4) $$k=\frac{H}{L}=cot\theta \cdot sinh^{-1}\left(tan\theta \right)=cot\theta \cdot ln\left(tan\theta +sec\theta \right)$$

(5) $$L=\left(N+1\right)\cdot S$$

(6) $$H=\frac{S}{V_{s}}\cdot \left(N+1\right)\cdot V_{b}$$

where *D_j_* denotes the theoretical hook depth (m); *H_j_* represents the horizontal distance between the hook and the nearest float; *F* is the float line length (m); *B* stands for the branch line length (m); *L* denotes the main line length between two adjacent floats; *N* is the number of branch lines per basket (16 in total); *k* represents the shrinkage rate; *θ* denotes the angle (°) between the tangent of the longline main line at the junction point of the float line end and the horizontal direction; *S* is the main line length between two adjacent branch lines (m); and vs. and *V_b_*, respectively, represent the line deployment speed and vessel speed (m/s).

#### 2.3.3. Local Spatial Autocorrelation

The local spatial autocorrelation Getis-Ord Gi* method (hot spot analysis method) can reveal the spatial agglomeration characteristics of attributes in local regions, identify statistically significant spatial clusters of high values (hot spots) and low values (cold spots), and calculate the spatial locations of clusters of high (or low) yields in tuna fishing grounds. The calculation formula for Getis-Ord Gi* is as follows [26]:

(7) $${Gi}^{*}=\frac{\sum_{j=1}^{n}w_{i,j}x_{j}-\overset{-}{X}\sum_{j=1}^{n}w_{i,j}}{s\times \sqrt{n\left(\sum_{j=1}^{n}w_{i,j}^{2}-{\left(\sum_{j=1}^{n}w_{i,j}\right)}^{2}\right)/\left(n-1\right)}}$$

where *x_j_* denotes the attribute value of *j* feature; *w_i,j_* represents the spatial weight between features *i* and *i* (1 for adjacent features and 0 for non-adjacent ones), in this study, the distance threshold was set based on the Queen contiguity rule for latitude-longitude grids; *n* is the total number of sample points. The G_i_* statistic yields a Z-score: if the Z-score is positive and significant, it indicates a hot spot, with a larger value representing a more concentrated aggregation of hot spots; if the Z-score is negative and significant, it indicates a cold spot, with a smaller value indicating a more concentrated aggregation of cold spots [27]. To ensure the significance of spatial distribution pattern aggregation, points with a Z-score not less than the 95% confidence level threshold were selected as hot spots, followed by interpolation analysis to identify the sea areas with hot spots of resource density for *T. obesus* and *T. albacares*.

#### 2.3.4. Statistical Analysis

Common correlation coefficients include the Pearson correlation coefficient, Spearman correlation coefficient, and Kendall rank correlation coefficient, among which the most commonly used are the Pearson and Spearman correlation coefficients. The former requires the data to meet three conditions simultaneously: (1) bivariate normal distribution; (2) continuous data; and (3) the existence of a linear relationship [28,29]. Shapiro–Wilk (S-W) tests performed on the data collected in this study revealed that all *p*-values were less than 0.05, which shows that the influence factors do not conform to a normal distribution. Thus, the Spearman correlation test was adopted to examine the correlations between tuna CPUE and various influencing factors, whose expression is as follows:

(8) $$\rho =1-\frac{6\sum {d_{i}}^{2}}{n\left(n^{2}-1\right)}\;$$

where *ρ* denotes the correlation coefficient; *d_i_* represents the rank difference between the corresponding data of the two variables; and *n* is the sample size. The closer the absolute value of the correlation coefficient between the two variables is to 1, the stronger the correlation between them, and the closer it is to 0, the weaker the correlation.

## 3. Results

### 3.1. The Hook Status of Tuna

**Figure 3:**
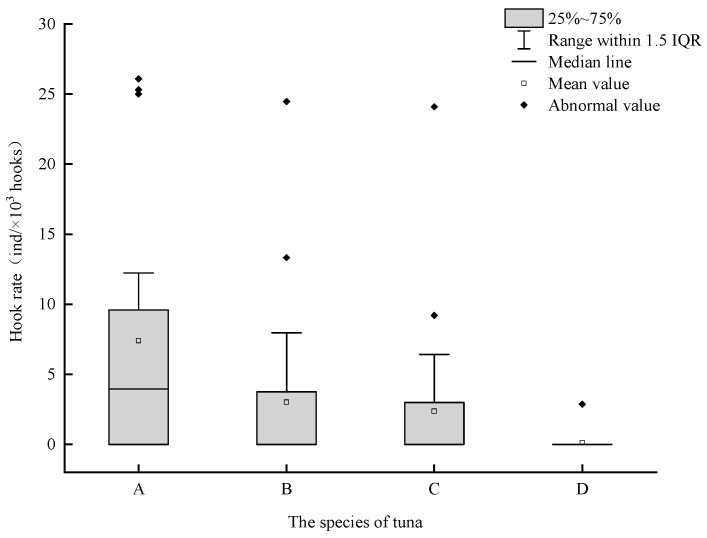
Hook rate for tuna in the high seas of the NWIO. Note: A, B, C, D represent *T. obesus* in 2023/2204, *T. albacares* in 2023/2024, *T. obesus* in 2024/2025, and *T. albacares* in 2024/2025, respectively.

Overall, the hook rate in 2023/2024 was higher than that in 2024/2025, and the hook rate of *T. obesus* was higher than that of *T. albacares* (Figure 3). In 2023/2024, a total of 32 *T. obesus* and 18 *T. albacares* were caught, with CPUE ranges of 0 to 25.32 ind/10^3^ hooks and 0 to 24.46 ind/10^3^ hooks, respectively; the average hook rates of the two survey voyages were 4.82 ind/10^3^ hooks and 2.71 ind/10^3^ hooks, respectively. In 2024/2025, 17 *T. obesus* and 1 *T. albacares* were captured in total, with CPUE ranges of 0 to 24.01 ind/10^3^ hooks and 0 to 2.87 ind/10^3^ hooks, respectively; the average hook rates of the two survey voyages were 2.33 ind/10^3^ hooks and 0.14 ind/10^3^ hooks, respectively.

### 3.2. The Geographical and Vertical Distributions of Tuna

**Figure 4:**
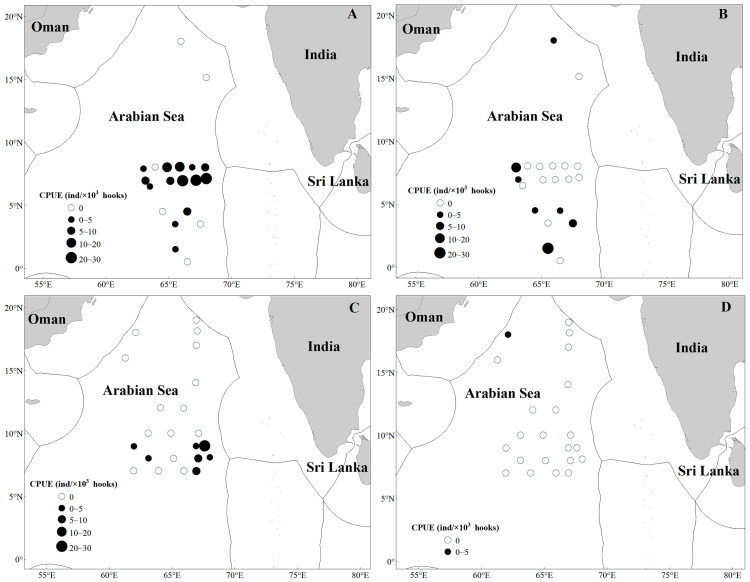
Distribution of Tuna CPUE in the high seas of the NWIO. Note: (**A**–**D**) represent *T. obesus* in 2023/2204, *T. albacares* in 2023/2024, *T. obesus* in 2024/2025, and *T. albacares* in 2024/2025, respectively.

The distribution of tuna CPUE is shown in Figure 4. *T. obesus* is mainly concentrated in the range of 63° E–69° E, 7° N–9° N; *T. albacares* exhibits a random distribution pattern with a wider distribution range than *T. obesus*, being relatively concentrated in 63° E–66° E, 1.5° N–5° N, and also occurring near 62° E–65° E, 18° N.

**Figure 5:**
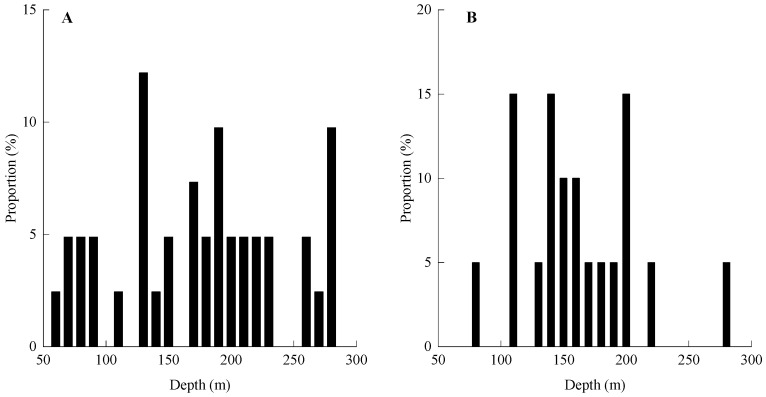
Distribution of *T. obesus* (**A**) and *T. albacares* (**B**) across Depth Strata in the high seas of the NWIO. Note: The data for A and B presented herein each include the datasets from the two cruises, respectively.

The vertical distribution of tuna across different water layers is presented in Figure 5. *T. obesus* is distributed in the water layer range of 60–280 m, with the highest distribution frequency (12.20%) observed in the 130–140 m layer, followed by the 180–190 m and 270–280 m layers, both with a distribution frequency of 9.76%. *T. albacares* is distributed in the range of 80–280 m, with relatively high distribution frequencies (15% each) in the 110–120 m, 140–150 m, and 200–210 m layers. The preferred water layers of *T. obesus* are deeper than those of *T. albacares*.

### 3.3. Hotspot Analysis

**Figure 6:**
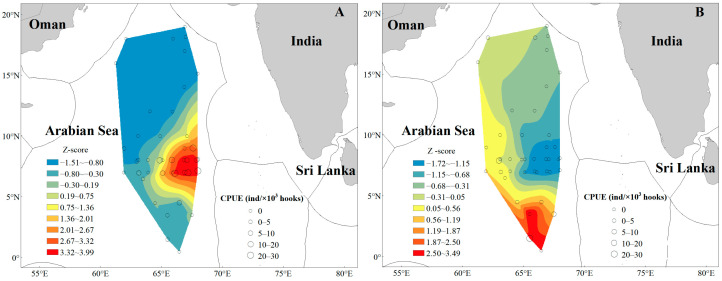
Hotspot analysis of *T. obesus* (**A**) and *T. albacares* (**B**) fishing areas in the high seas of the NWIO.

Overall, both *T. obesus* and *T. albacares* exhibited distinct aggregation characteristics in their spatial distribution, with the presence of concentrated hotspot sea areas. The hotspot areas of *T. obesus* were mainly clustered in the waters ranging from 63° E to 69° E and 5° N to 10° N, while those of *T. albacares* were primarily concentrated in the region between 64° E and 68° E and 0° N to 4° N (Figure 6). The mean CPUEs of *T. obesus* and *T. albacares* in their respective hotspot areas were 11.72 ind/10^3^ hooks and 6.71 ind/10^3^ hooks, respectively, whereas their mean CPUE values in non-hotspot areas were 3.41 ind/10^3^ hooks and 0.64 ind/10^3^ hooks.

### 3.4. Correlation Analysis

The results of the correlation analysis showed that CPUEdy exhibited a highly significant negative correlation with latitude and Abuzoo (*p* < 0.01), while CPUEhq had no significant correlation with longitude, latitude, or plankton (*p* > 0.05) (Table 1).

The correlations between environmental variables across different water layers and CPUEdy as well as CPUEhq are presented in Table 2. CPUEdy exhibited highly significant positive correlations with ST5, ST25, and ST50 (*p* < 0.01). In contrast, CPUEhq showed a highly significant positive correlation with ST75 (*p* < 0.01) and a highly significant negative correlation with ST200 (*p* < 0.01). For chlorophyll-a (Chl-a) concentrations, CPUEdy was highly significantly negatively correlated with Chl-a5 (*p* < 0.01), significantly positively correlated with Chl-a75 (*p* < 0.05), and highly significantly positively correlated with Chl-a100, Chl-a150, Chl-a200, and Chl-a300 (*p* < 0.01). Meanwhile, CPUEhq was significantly positively correlated with Chl-a50 (*p* < 0.05) and highly significantly positively correlated with Chl-a100 (*p* < 0.01). Regarding salinity (Sal), CPUEdy displayed highly significant negative correlations with Sal in all water layers except Sal75 (*p* < 0.01). CPUEhq was highly significantly negatively correlated with Sal5 (*p* < 0.01) and significantly negatively correlated with Sal25, Sal50, Sal75, Sal100, and Sal300 (*p* < 0.05).

### 3.5. The Relationship Between the Distribution of Tuna Resources and Environmental Factors

**Figure 7:**
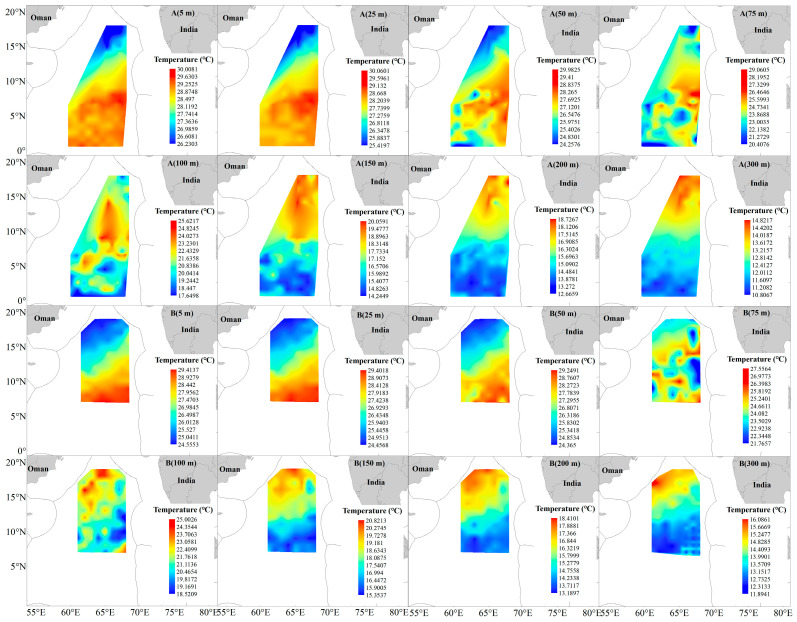
Water temperature at different depths in the survey area of the NWIO during 2023/2024 (**A**) and 2024/2025 (**B**).

In the tuna fishing grounds surveyed during the 2024/2025 cruise, sea temperatures at 5 m (ST5) and 25 m (ST25) decreased significantly compared with those in 2023/2024, whereas sea temperatures at other depths showed no statistically significant variations. The average ST5 dropped from 28.99 °C to 27.08 °C, and average ST25 decreased from 28.92 °C to 26.99 °C. In terms of spatial distribution, shallow water temperatures (<25 m) exhibited a south-high, north-low pattern, while deep water temperatures (>50 m) showed the opposite trend (north-high, south-low) (Figure 7). CPUE analysis revealed a significant positive correlation between CPUE and SST for both *T. obesus* and *T. albacares*, indicating a preference for warmer surface waters (Table 2, Figure 4).

**Figure 8:**
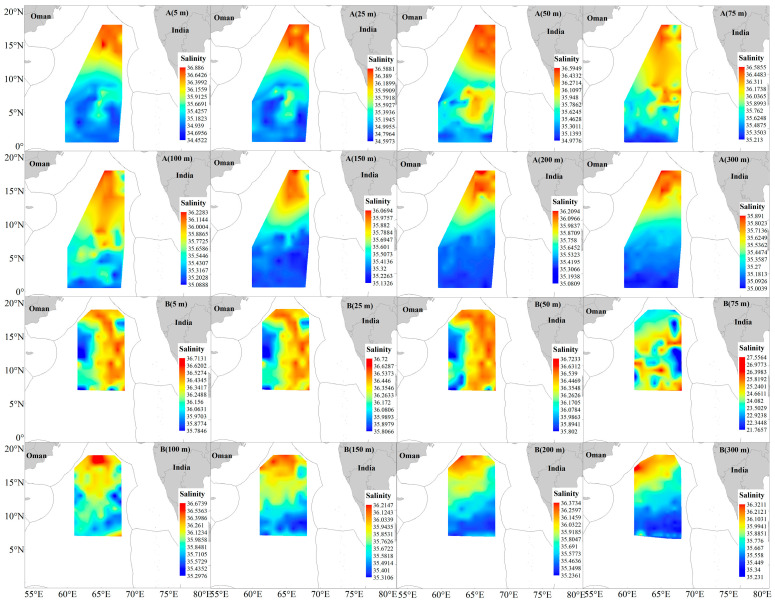
Salinity at different depths in the survey area of the NWIO during 2023/2024 (**A**) and 2024/2025 (**B**).

In 2023/2024, salinity across all water layers displayed a north-high, south-low spatial pattern. However, the salinity distribution changed markedly in 2024/2025: a distinct east–west gradient (east-high, west-low) emerged in the 5–50 m water layer, while the north-high, south-low pattern was retained in waters deeper than 100 m (Figure 8). CPUE analysis indicated a significant negative correlation between catch per unit effort and salinity for both tuna species (Table 2, Figure 4), suggesting that they favor low-salinity waters.

**Figure 9:**
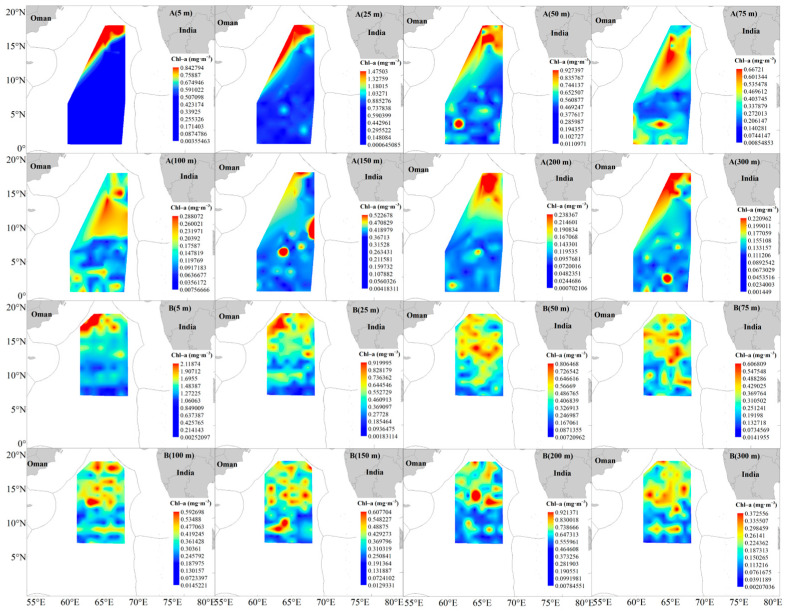
Chl-a at different depths in the survey area of the NWIO during 2023/2024 (**A**) and 2024/2025 (**B**).

In 2023/2024, Chl-*a* concentrations across all water layers followed a north-high, south-low distribution. The 2024/2025 Chl-*a* distribution exhibited novel characteristics: the pattern in waters shallower than 25 m reversed to south-high, north-low, while pronounced spatial heterogeneity was observed in other water layers (Figure 9). CPUE analysis revealed that the two tuna species exhibited a preference for surface waters with low Chl-*a* concentrations and deep waters with high Chl-*a* concentrations.

## 4. Discussion

Significant differences in CPUE were observed between the two survey cruises for both *T. obesus* and *T. albacares*. The CPUE of both species during the 2023/2024 cruise was significantly higher than that in 2024/2025, and *T. obesus* consistently exhibited higher CPUE than *T. albacares* (Figure 2). This phenomenon may be ascribed to multiple factors, with changes in SST being a key driver. Studies have demonstrated that global SST increased abnormally during 2023–2024, with the average SST 0.25 °C higher than that in 2015–2016 [30], and returned to a normal warming trend in 2024–2025 (Figure 7). Research indicates that both *T. obesus* and *T. albacares* tend to aggregate in relatively warm waters [7], a behavior closely linked to the role of warm waters as primary habitats for mesopelagic squids, teleosts, crustaceans, and other marine organisms [31,32]. Specifically, warm waters not only offer a suitable habitat for these prey species but also may indirectly enhance the foraging efficiency of tuna and reinforce their habitat preference by increasing the population density and activity of prey. Thus, the abnormally high temperatures in 2023/2024 may be a key factor contributing to the significantly higher tuna CPUE compared to 2024/2025.

However, it should be noted that while sea temperature may be one of the primary factors influencing CPUE in the two cruises, the differences in the survey areas between the cruises also constitute a significant cause of CPUE variations. In the western Indian Ocean, yellowfin tuna and skipjack tuna (*Katsuwonus pelamis*) represent the predominant catch species, followed by swordfish (*Xiphias gladius*) and bigeye tuna [33]. Nevertheless, our survey indicates that the catch of bigeye tuna substantially exceeds that of yellowfin tuna (Figure 3), a result attributable to the selected survey areas. In the northern Indian Ocean, the fishing grounds for yellowfin tuna are primarily distributed between 0° N and 10° N, with hotspot concentrations occurring between 0° N and 5° N [34]. Consequently, the disparities between the cruise stations likely constitute the main reason for the significant variations in yellowfin tuna catches.

In addition, the inherent interannual variability in tuna resource abundance constitutes another undeniable contributing factor. Studies have demonstrated that tuna abundance in the Indian Ocean exhibits natural fluctuations, which may be linked to climate indices including the IOD [35,36] and the Madden-Julian Oscillation (MJO) [37]. Setiawati et al. [9] reported that peak tuna catches in the eastern Indian Ocean off Java-Bali coincided with positive IOD events. Research has also indicated that the Indian Ocean is subject to concurrent influences of both ENSO and IOD [38,39].

The spatial and vertical distributions of *T. obesus* and *T. albacares* exhibit distinct differences: the former has a broader vertical distribution range but a relatively concentrated horizontal distribution, whereas the latter occupies a narrower vertical water column yet features a wider horizontal distribution (Figure 4). In comparison, *T. obesus* exhibits a more stable catch rate [10], a pattern that may be directly associated with its relatively clustered spatial distribution. Previous studies have demonstrated that the fishing grounds of these two species display obvious seasonal variability, with significant differences in the geographic centers of their fishing grounds [10,40]—a characteristic that may explain the marked disparities in their horizontal distributions. The northern Indian Ocean is characterized by a typical monsoon-driven climate, with the northeast monsoon prevailing in winter and the southwest monsoon dominating in summer [41,42]. In summer, upwelling induced by the Somali Cold Current significantly enhances plankton reproduction, providing abundant forage for fish resources [43] and thereby promoting the formation of seasonal fishing grounds. Meanwhile, monsoon currents and warm current regions in this area offer suitable habitats and spawning environments for various fish species, including tuna and skipjack tuna, making it a key area for the frequent activities of multiple migratory fish species. All survey periods in this study were conducted selectively during December, January, and February of the following year—a period that coincides with the northeast monsoon season in the northern Indian Ocean (December to April of the subsequent year) [41,42]. During this period, ocean currents in the region flow counterclockwise, forming a circulation system consisting of the southward Somali Warm Current along the Somali Peninsula coast, the Equatorial Countercurrent near the equator, and monsoon currents in the Arabian Sea [40]. Therefore, we hypothesize that the southward Somali Warm Current may be one of the critical drivers shaping the migratory distribution of these two tuna species during this season.

Both *T. obesus* and *T. albacares* exhibit diel vertical migration behavior [16,20,21]. Studies have demonstrated that *T. obesus* displays a strong ability to penetrate the thermocline, with its daytime activity range extending as deep as 400–500 m [20], whereas *T. albacares* predominantly inhabits waters between 50 and 300 m [21]. This vertical stratification acts as a critical strategy to reduce interspecific competition: *T. obesus* is more adapted to the low-temperature, high-dissolved oxygen environment of deep waters, while *T. albacares* is more sensitive to the thermohaline conditions of the shallower epipelagic and mesopelagic zones. Yang [22] noted that, in comparison, *T. obesus* prefers low-temperature and low-salinity waters, whereas *T. albacares* favors high-temperature and high-salinity waters. However, it should be emphasized that the resource abundances of both tuna species show a significant negative correlation with salinity (Sal) (Table 2). Thus, the conclusion that *T. albacares* prefers high-salinity waters is relative—i.e., in comparison to *T. obesus*. In addition, differences in their vertical distributions may also be linked to dietary differentiation. Research indicates that the feeding depth of *T. obesus* is primarily 100–300 m (i.e., the epipelagic and mesopelagic layers) [44,45,46], while the prey of *T. albacares* almost exclusively inhabit the surface or epipelagic zones [47,48,49]. This also explains the phenomenon observed in this study: *T. obesus* has a high catch rate in deep water layers, whereas catches of *T. albacares* mostly occur in waters shallower than 200 m (Figure 5). Nevertheless, the internal regulatory mechanisms underlying the habitat preferences of the two species remain to be further investigated.

This study used common environmental variables (ST, Chl-a, Sal, DO) to examine their correlations with bigeye and *T. albacares*, all of which are considered important factors influencing the habitats of both species [15,50,51]. Research has shown that bigeye and *T. albacares* are often active above, near, or below the thermocline [8], and changes in environmental factors across different water layers are crucial for their distribution and density.

Compared with the 2023/2024 cruise, the average values of ST5 and ST25 in the 2024/2025 cruise decreased significantly (by 1.91 °C and 1.93 °C, respectively) (Figure 7), and the CPUE of both *T. obesus* and *T. albacares* plummeted over the same period. The decline in SST may have driven a southward shift in the centroids of tuna fishing grounds. Previous studies have indicated that SST is a key environmental variable affecting tuna habitat selection [52,53], a conclusion supported by the results of this study: the CPUE of *T. obesus* exhibited highly significant positive correlations with ST5 (*r* = 0.688), ST25 (*r* = 0.641), and ST50 (*r* = 0.545) (all *p* < 0.01) (Table 2). The findings of this study suggest that temperature in the shallow 5–50 m water layer is a critical variable driving variations in *T. obesus* abundance in the northwestern Indian Ocean; specifically, habitat suitability for *T. obesus* decreases when the shallow water temperature drops below 28 °C. Song et al. [54] reported that temperature at the 200 m depth is an important variable for predicting *T. albacares* fishing grounds, which is consistent with the results of this study—i.e., the abundance of *T. albacares* was significantly correlated with ST200 (*r* = −0.714, *p* < 0.05). As key factors regulating the primary productivity of marine ecosystems, chlorophyll-*a* (Chl-*a*), salinity (Sal), and dissolved oxygen (DO) also often exert significant impacts on the distribution of fishery resources. In this study, SST, Chl-*a* and Sal all influenced the resource abundance of both tuna species, with their effects varying markedly across different water layers. Meanwhile, the two species also exhibited significant differences in their responses to environmental factors across distinct water layers (Table 2). Environmental data derived from different sources may lead to divergent research results: for instance, based on remote sensing data analysis, Xu et al. [10] and Wang [51] argued that Chl-*a* shows no obvious correlation with the distribution of *T. obesus* and *T. albacares*, whereas Chen [55] concluded that Chl-*a* exerts a key influence on the distribution of *T. obesus* fishing grounds and noted that the optimal Chl-*a* concentration varies seasonally.

*T. obesus* and *T. albacares* exhibit significant differences in their responses to environmental factors, reflecting their distinct survival and adaptation strategies. However, as highly migratory species, more attention should be paid to their migration characteristics and environmental preferences.

This study is primarily based on fisheries scientific survey data from two cruises, and the dataset size is relatively limited. In addition, the discrepancy in sampling stations between the two cruises, while expanding the survey coverage, introduces a certain degree of randomness into the survey results. Meanwhile, our surveys were only conducted from December to February of the following year, and solely focused on longline surveys without incorporating other fishing methods, which has a certain impact on the generalizability of the results. Therefore, in future research, it is essential not only to increase the number of sampling stations and incorporate other fishing gears to accumulate a more comprehensive dataset, but also to consider the impacts of factors such as ENSO, IOD, and sea surface height on *T. obesus* and *T. albacares*, so as to conduct more in-depth research on these species.

## 5. Conclusions

Based on data from two fishery scientific surveys, this study investigated the spatiotemporal and vertical distribution patterns of Thunnus obesus and Thunnus albacares, along with their correlations with environmental factors, in the high seas of the NWIO. Results showed that *T. obesus* had significantly higher catch and hook rates than *T. albacares* across the survey area. While *T. albacares* exhibited a wider horizontal distribution range, *T. obesus* occupied a more extensive vertical water column. Distribution hotspots of *T. obesus* were located north of those of *T. albacares*, which concentrated near equatorial waters; resource abundance in hotspots was statistically higher than in non-hotspot areas. These interspecific distributional disparities are hypothesized to alleviate interspecific food competition. Further, *T. albacares* showed a stronger preference for warm epipelagic and mesopelagic waters than *T. obesus*, with both species favoring low-salinity marine environments.

Despite using rigorous scientific survey data to clarify the core distributional traits, environmental preferences, and key ecological phenomena of *T. obesus* and *T. albacares*, this study has inherent limitations, chiefly stemming from limited survey cruises and sampling stations. This constraint hindered detailed analysis of the seasonal dynamics of tuna stocks. Future research should thus prioritize investigating the migratory dynamics of tuna in the NWIO and the mechanisms underlying their adaptive responses to climate change.

## Figures and Tables

**Table 1:** Correlation analysis between the CPUE of *T. obesus* and *T. albacares* with geographical and planktonic factors in the high seas of the NWIO.

Influence Factor	Longitude	Latitude	Abuzoo	Abuphy
CPUEdy	0.221	−0.424 **	−0.624 **	−0.286
CPUEhq	−0.170	−0.295	−0.269	−0.078

Note: ** indicates *p* < 0.01.

**Table 2:** Correlation analysis between the CPUE of *T. obesus* and *T. albacares* with environmental factors at different depth layers in the high seas of the NWIO.

Influence Factor	Tuna	5 m	25 m	50 m	75 m	100 m	150 m	200 m	300 m
ST	CPUEdy	0.668 **	0.641 **	0.545 **	0.22	0.156	−0.218	−0.248	−0.292
	CPUEhq	0.333	0.262	0.286	0.214 **	−0.143	−0.667	−0.714 *	−0.619
Chl-a	CPUEdy	−0.399 **	−0.162	0.197	0.335 *	0.483 **	0.460 **	0.462 **	0.339 **
	CPUEhq	−0.108	0.049	0.379 *	0.3	0.400 **	0.275	0.28	0.350 **
Sal	CPUEdy	−0.396 **	−0.428 **	−0.409 **	−0.155	−0.312 **	−0.492 **	−0.435 **	−0.496 **
	CPUEhq	−0.367 **	−0.305 *	−0.318 *	−0.373 *	−0.319 *	−0.238	−0.302	−0.329 *

Note: ** indicates *p* < 0.01; * indicates *p* < 0.05.

## Data Availability

The original contributions presented in the study are included in the article, and further inquiries can be directed to the corresponding authors.
